# Supplementary material for: Functional Analysis of KAP1/TRIM28 Requirements for HIV-1 Transcription Activation
Source: Viruses. 2024 Jan 13;16(1):116. doi: 10.3390/v16010116 (PMC10819576; doi:10.3390/v16010116)
Supplement: Supplementary file 1 [file viruses-16-00116-s001.zip › viruses-2673500-supplementary.pdf]

Supplementary Materials

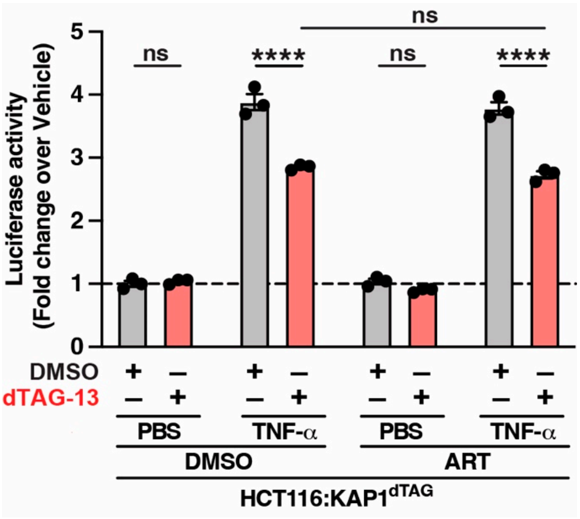

Supplementary Figure S1. Acute KAP1 depletion equally decreases signal-induced HIV-1 reporter activation in the absence and presence of ART. Luciferase assay in the HCT116:KAP1^{dTAG} cell line transiently transfected with the HIV-1 FFL and pRL-Null luciferase reporters for 16 h. Cells were pre-treated with dTAG-13 or Vehicle (DMSO) for 8 h, then treated with ART or Vehicle (DMSO) for 16 h and finally challenged with TNF-α or Vehicle (PBS) for 4 h after ART. Luciferase activity is plotted as Fold change over Vehicle DMSO (line 1). Data represent mean +/– SEM. Fold luciferase activity (N = 3, two-way ANOVA followed by Sidak's test for multiple comparisons). **** p <0.0001, and ns = non-significant.

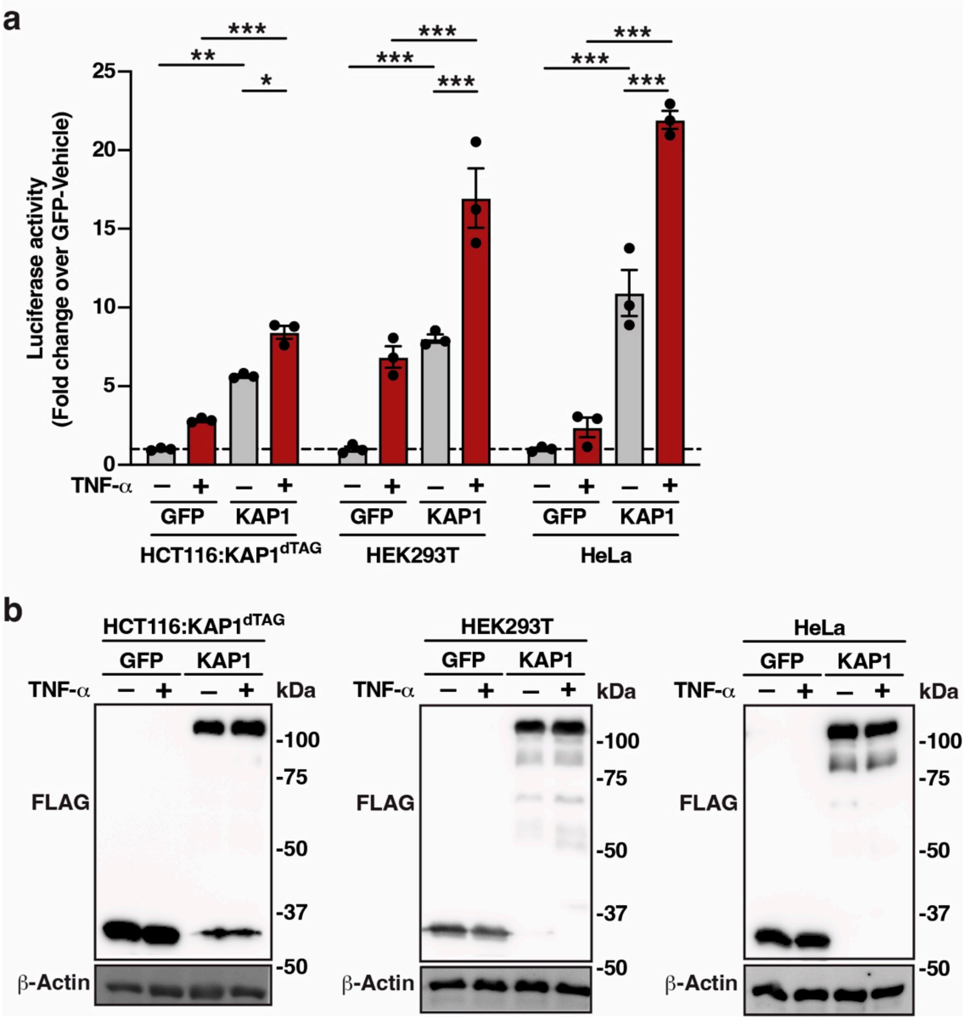

Supplementary Figure S2. Ectopic KAP1 activates the HIV-1 reporter in a cell-type independent manner and TNF-α has an additive effect. (**a**) Luciferase assay in the indicated cell lines transiently transfected with the HIV-1 FFL and pRL-Null luciferase reporters for 24 h alongside GFP and KAP1 expressing plasmids followed by 4 h treatment with TNF-α (+) or vehicle (PBS) (–). Luciferase activity is plotted as Fold change over GFP-Vehicle (PBS) condition (line 1). Data represent mean +/– SEM. Fold luciferase activity (N = 3, two-way ANOVA followed by Sidak's test for multiple comparisons). * $p < 0.05$, ** $p < 0.01$, *** $p < 0.001$, and ns = non-significant. (**b**) Western blot analysis of the samples indicated in panel (a). Blots are representative of three independent experiments.

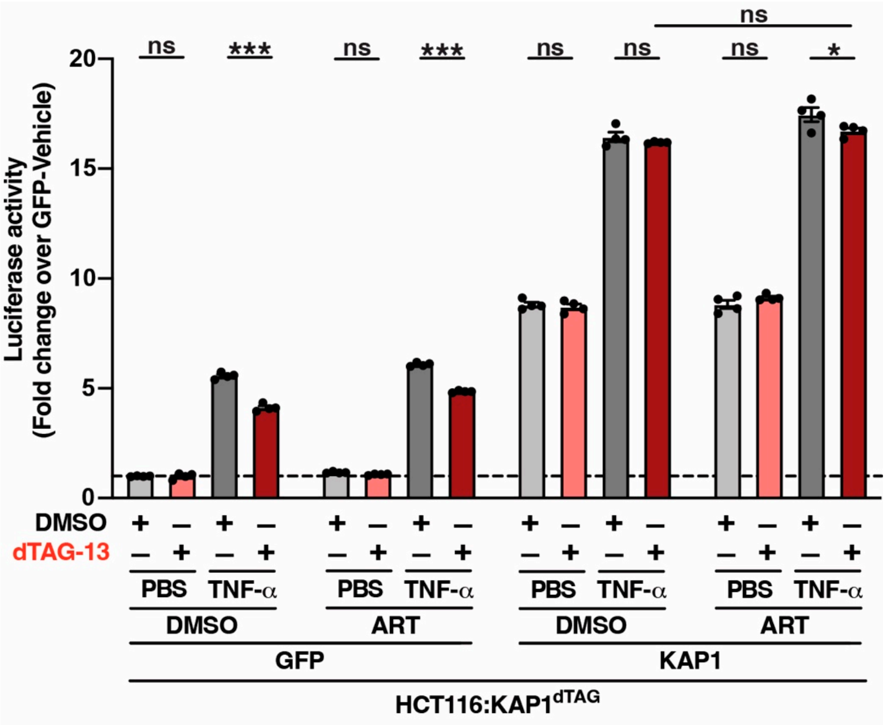

Supplementary Figure S3. The presence of ART does not impact activation of the HIV-1 promoter by KAP1 and potentiation by TNF-α. Luciferase assay in the HCT116:KAP1^{dTAG} cell line transiently transfected with the HIV-1 FFL and pRL-Null luciferase reporters for 24 h alongside GFP or KAP1 expressing plasmids. Cells were treated with dTAG-13 or Vehicle (DMSO) for 8 h followed by treatment with ART for 16 h. Then cells were stimulated with TNF-α or Vehicle (PBS) for 4 h. Luciferase activity is plotted as Fold change over GFP-Vehicle (DMSO) condition (line 1). Data represent mean +/– SEM. Fold luciferase activity (N = 4, two-way ANOVA followed by Tukey's test for multiple comparisons). * $p < 0.05$, *** $p < 0.001$, and ns = non-significant.

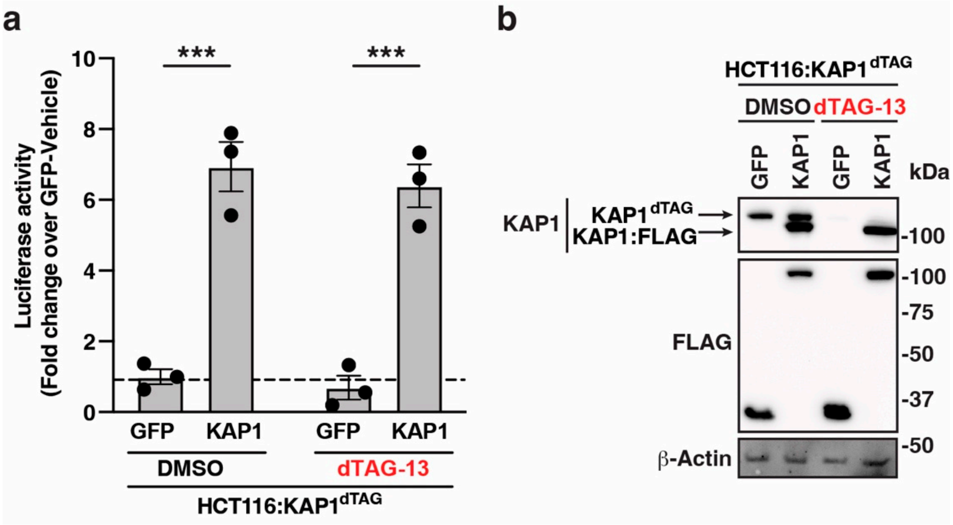

Supplementary Figure S4. Ectopic KAP1 equally activates the HIV-1 reporter in HCT116:KAP1^{dTAG} cells in the absence and presence of dTAG-13. (**a**) Luciferase assay in the HCT116:KAP1^{dTAG} cell line transiently co-transfected with the HIV-1 FFL and pRL-Null luciferase reporters alongside GFP or KAP1 expressing plasmids for 16 h, and treated with DMSO or dTAG-13 for 24 h. Luciferase activity is plotted as Fold change over GFP-Vehicle (DMSO) condition (line 1). Data represent mean +/– SEM. Fold luciferase activity (N = 3, two-way ANOVA followed by Tukey's test for multiple comparisons). *** $p < 0.001$. (**b**) Western blot analysis of the samples from panel (a). Blots are representative of three independent experiments.

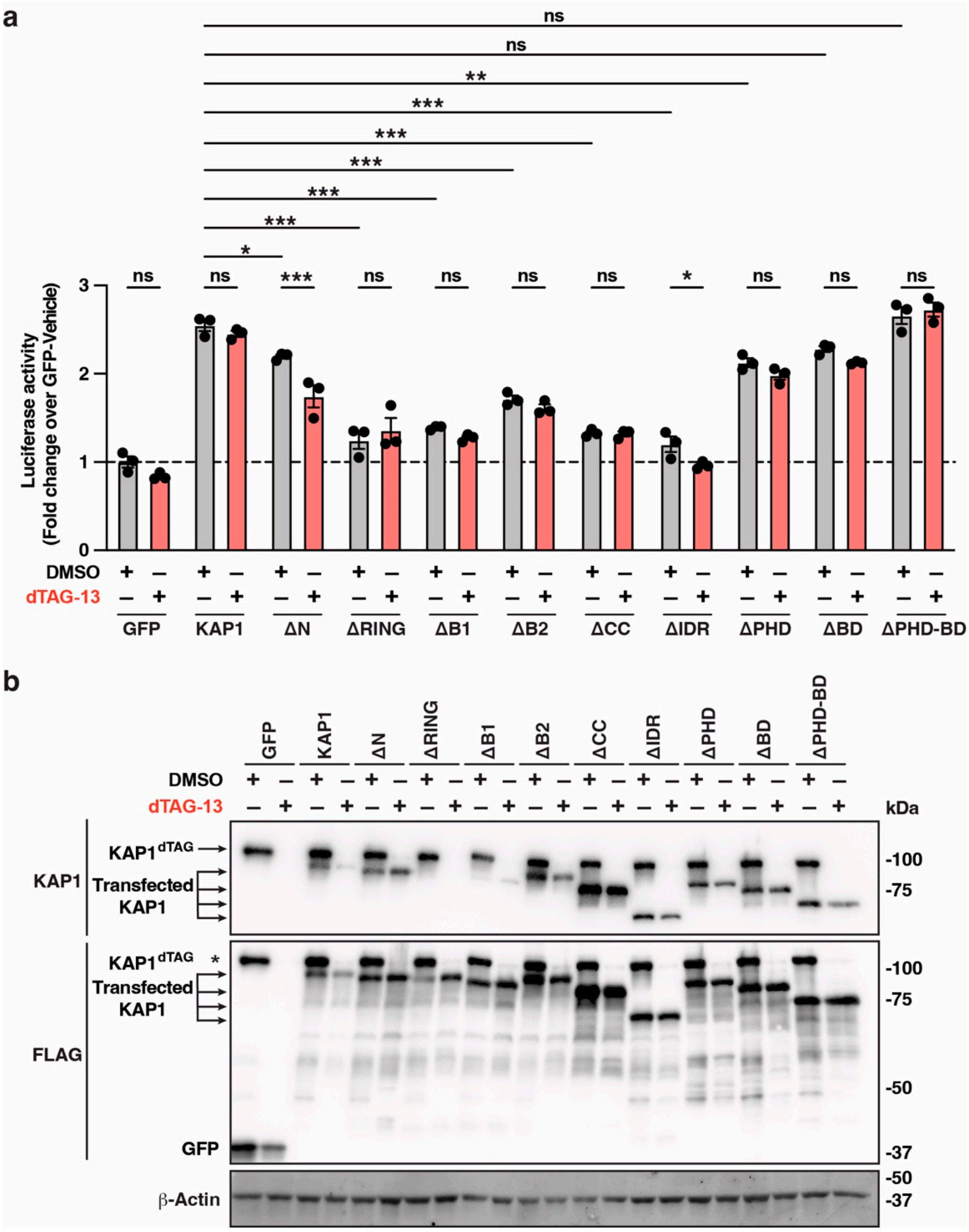

Supplementary Figure S5. The presence of endogenous KAP1 does not largely alter the activation of the HIV-1 promoter by the ectopically expressed KAP1 constructs. (**a**) Fold luciferase activity comparing over-expression of GFP and KAP1 (full length or deletion constructs) in HCT116:KAP1^{dTAG} cells treated with DMSO or dTAG-13 for 24 h. Luciferase activity is plotted as Fold change over GFP-Vehicle (DMSO) condition (line 1). Data represent mean +/– SEM. Fold luciferase activity (N = 3, two-way ANOVA followed by Sidak's test for multiple comparisons). * *p* < 0.05, ** *p* <0.01, *** *p* < 0.001, and ns = non-significant. (**b**) Western blot analysis of samples from panel (a). The top membrane was first probed with the anti-KAP1 antibody, then stripped, and re-probed with the anti-FLAG antibody. The asterisk (*) in the FLAG blot represents the signal of endogenous KAP1^{dTAG} remaining from an imperfectly stripped anti-KAP1 blot. Blots are representative of three independent experiments.

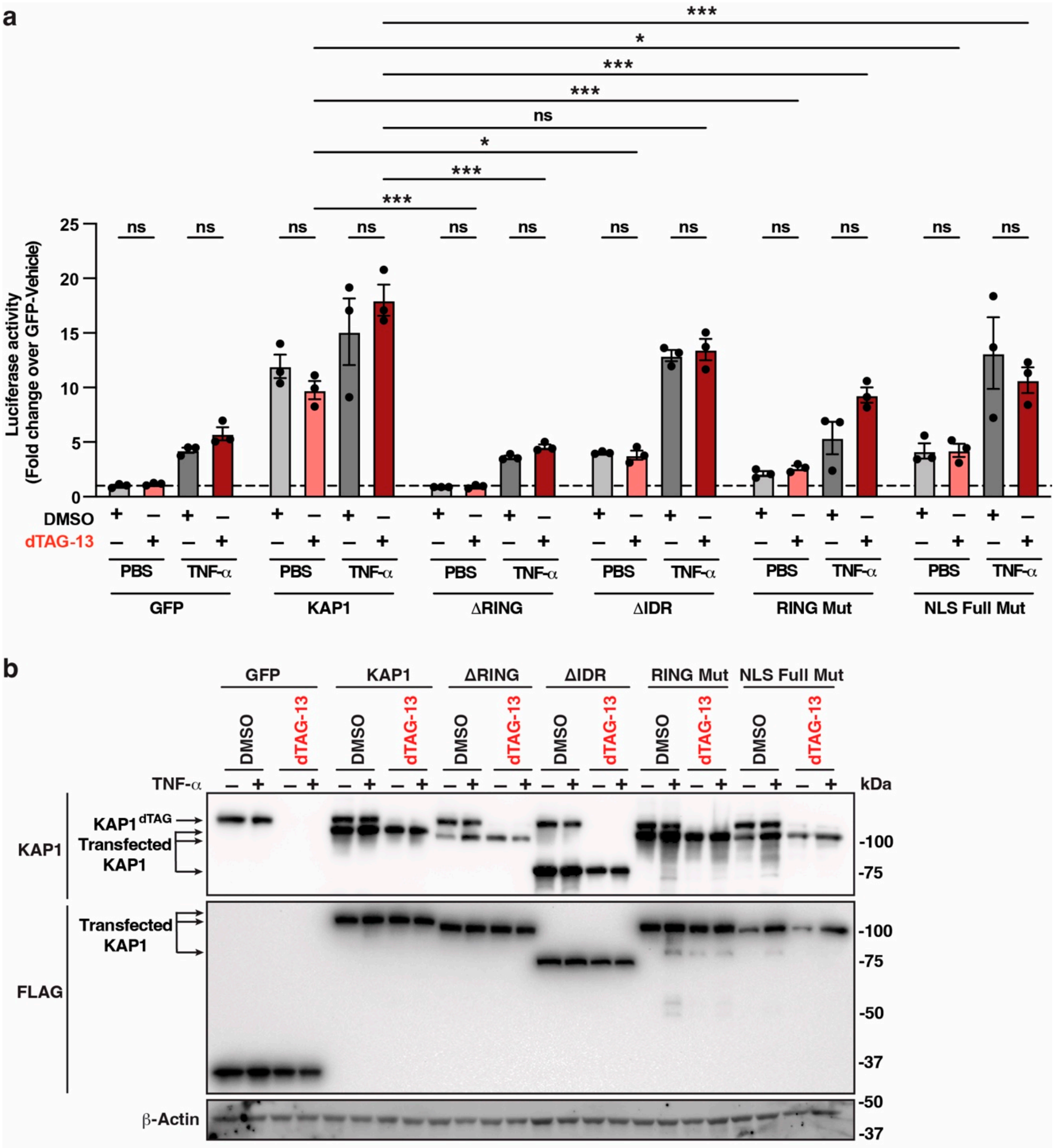

Supplementary Figure S6. The KAP1 non-functional mutants do not largely alter signal-induced HIV-1 reporter activation by endogenous KAP1. (**a**) Fold luciferase activity comparing over-expression of GFP and KAP1 (full length, ΔRING, ΔIDR, RING Mut, or NLS Full Mut) in HCT116:KAP1^{dTAG} cells treated with DMSO or dTAG-13 for 24 h followed by treatment with TNF-α or vehicle (PBS) for 4 h. Luciferase activity is plotted as Fold change over GFP-Vehicle (DMSO) condition (line 1). Data represent mean +/– SEM. Fold luciferase activity (N = 3, two-way ANOVA followed by Tukey's test for multiple comparisons). * $p < 0.05$, ** $p < 0.01$, *** $p < 0.001$, and ns = non-significant. (**b**) Western blot analysis of samples from panel (a). Blots are representative of three independent experiments.

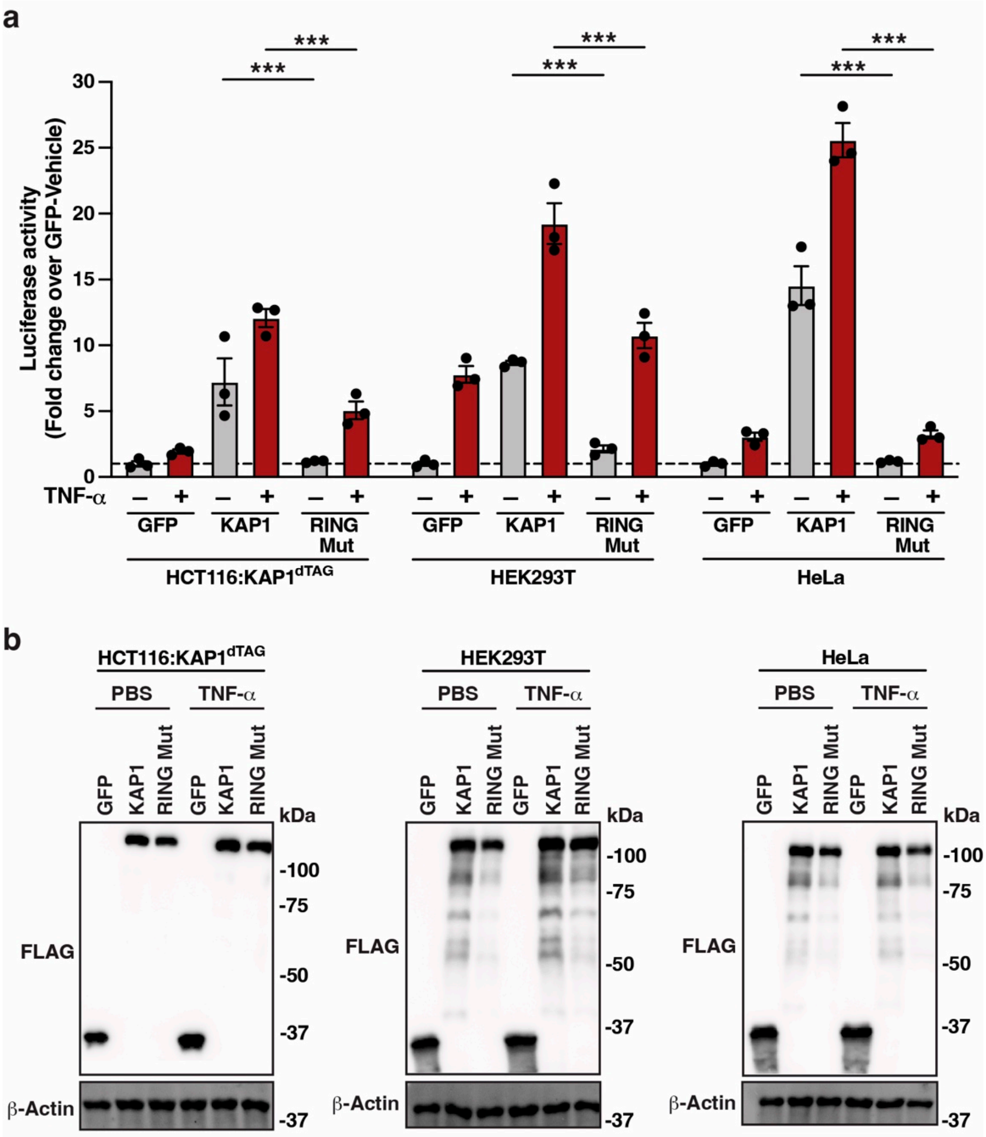

Supplementary Figure S7. The KAP1 RING mutant does not largely alter signal-induced HIV-1 reporter activation by endogenous KAP1. (**a**) Luciferase assay comparing ectopic expression of GFP and KAP1 in the indicated cell lines. Following 24 h transfection, cells were treated with TNF-α or vehicle (PBS) for 4 h. Luciferase activity is plotted as Fold change over GFP-Vehicle (PBS) condition (line 1). Data represent mean +/– SEM. Fold luciferase activity (N = 3, two-way ANOVA followed by Sidak's test for multiple comparisons). *** $p < 0.001$. (**b**) Western blot analysis of samples from panel (a). Blots are representative of three independent experiments.

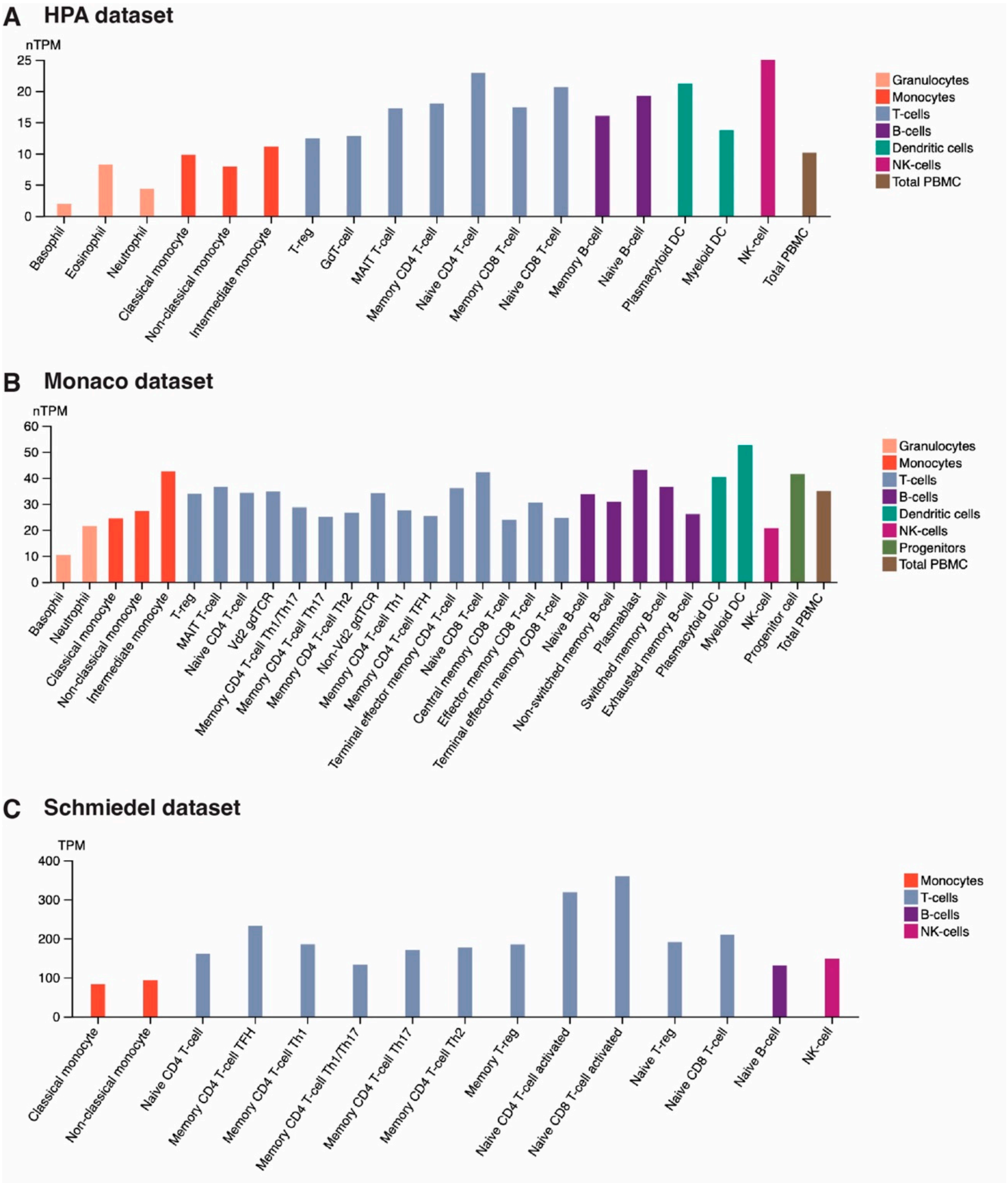

Supplementary Figure S8. KAP1 expression levels in human immune cells from publicly available resources. (**a**) KAP1 transcript expression values calculated as nTPM (normalized Transcripts Per Million), resulting from the internal normalization pipeline for 18 immune cell types and total peripheral blood mononuclear cells (PBMCs) from the Human Protein Atlas (HPA) dataset. (**b**) KAP1 nTPM resulting from the internal normalization pipeline visualized for 29 blood cell types and total PBMCs from the Monaco dataset [49]. (**c**) KAP1 TPM values visualized for 15 blood cell types from the Schmiedel dataset [50].

Supplementary Table S1. DNA plasmids used in this study.

Plasmid	Description [Reference]	Primers used (assay)
pcDNA3.1-HIV-1–LTR-FFL	[41]	NA
pBluescript II KS+	Agilent	NA
pRL-Null	Promega, E2271	NA
pRL-TK	Promega, E2241	NA
pGL3-3xAP1-FFL	Addgene, 40342	NA
pGL3-NF-κB-FFL	[42]	NA
pGL3-NFAT-FFL	Addgene, 17870	NA
pTRIP-GFP	GFP	38: 5'-CCGACTAGTATGGTGAGCAAGGGC-3'<br>26: 5'-CCGCTCGAGCTACTTGTACAGCTC-3'<br>(PCR cloning)
pTRIP-KAP1	1-835 AA	1413: 5'-CCGACTAGTATGGCGGCCTCCGCGGCGGC-3'<br>1414: 5'-CCGCTCGAGTCAGGGGCCATCACCAGGGCCAC-3'<br>(PCR cloning)
psPAX2	Addgene, 12260	NA
pMD2.G	Addgene, 12259	NA
pcDNA4/TO-GFP:SF	GFP:SF	NA
pcDNA4/TO-KAP1:F	1-835 AA [26]	1107: 5'-CCGAAGCTTATGGCGGCCTCCGCGGCGGC-3'<br>1108: 5'-CCGCTCGAGGGGGCCATCACCAGGGCCAC-3'<br>(PCR cloning)
pcDNA4/TO-KAP1ΔN:F	53-835 AA [This study]	3890: 5'-CCGAAGCTTATGGGGGGCGGCGCCGAG-3'<br>1108: 5'-CCGCTCGAGGGGGCCATCACCAGGGCCAC-3'<br>(PCR cloning)
pcDNA4/TO-KAP1ΔRING:F	1-63 AA, 139-835 AA [This study]	3620: 5'-GGCAGCAAGGCTGCCACC-3'<br>3621: 5'-CTCCAGCAGCTCCAGCGC-3'<br>(PCR cloning)
pcDNA4/TO-KAP1ΔB1:F	1-138 AA, 204-835 AA [This study]	3622: 5'-GAACGTACTGTCTATTGC-3'<br>3623: 5'-ACTATCACGCATGAAATAATTC-3'<br>(PCR cloning)
pcDNA4/TO-KAP1ΔB2:F	1-203 AA, 244-835 AA [This study]	3624: 5'-TTCTTAGAGGATGCAGTGAGG-3'<br>3625: 5'-ACCATCCCGAGACTTGGC-3'<br>(PCR cloning)
pcDNA4/TO-KAP1ΔCC:F	1-245 AA, 377-835 AA [This study]	1755: 5'-AAGATGATTGTGGATCCC-3'<br>1756: 5'-TAAGAACTGGTACTGGTG-3'<br>(PCR cloning)
pcDNA4/TO-KAP1ΔIDR:F	1-420 AA, 625-835 AA [This study]	3626: 5' GCCACCATTTGCCGTGTC-3'<br>3627: 5'-AGGGCCTGTTGAGTTAGTG-3'<br>(PCR cloning)
pcDNA4/TO-KAP1ΔPHD:F	1-622 AA, 674-835 AA [This study]	<u>1-622 AA</u><br>1107: 5'-CCGAAGCTTATGGCGGCCTCCGCGGCGGC-3'<br>1748: 5'-CTCCTCCTTCAGGTCATCCAGGGTTCC-3'<br><u>674-835 AA</u><br>1749: 5'-CCGGGAACCCTGGATGACCTGAAGGAG-3'<br>1108: 5'-CCGCTCGAGGGGGCCATCACCAGGGCCAC-3'

		(PCR cloning)
pcDNA4/ TO-KAP1 ΔBD:F	1-696 AA<br>[This study]	1107: 5’-CCGAAGCTTATGGCGGCCTCCGCGGCGGC-3’<br>1751: 5’-AATCTCGAGCTTGGCCACCAC-3’<br>(PCR cloning)
pcDNA4/ TO-KAP1 ΔPHD-BD:F	1-622 AA<br>[This study]	1107: 5’-CCGAAGCTTATGGCGGCCTCCGCGGCGGC-3’<br>1750: 5’-AATCTCGAGGTCCAGGGTTCC-3’<br>(PCR cloning)
pcDNA4/ TO-KAP1 RING mutant:F	1-835 AA, C65A<br>[This study]	1738: 5’-GCTGCTGGAGCACGCCGGCGTGTGCAGA-3’<br>1739: 5’-TCTGCACACGCCGGCGTGCTCCAGCAGC-3’<br>(Site-directed mutagenesis)
pcDNA4/ TO-KAP1 B1 Box mutant:F	A160D/T163A/ E175R<br>[This study]	Primers to mutate A160D/T163A<br>3891: 5’-GCTGTGAGGATAATGACCCAGCCGCCAGCTACTGTG-3’<br>3892: 5’-CACAGTAGCTGGCGGCTGGGTCATTATCCTCACAGC-3’<br><br>Primers to mutate E175R<br>3893: 5’-CTCGGAGCCTCTGTGTAGGACCTGTGTAGAGGCG-3’<br>3894: 5’-CGCCTCTACACAGGTCCTACACAGAGGCTCCGAG-3’<br>(Site-directed mutagenesis)
pcDNA4/ TO-KAP1 CC mutant:F	V293S/K296A/M297 A/L300S<br>[This study]	Primers to mutate V293S/L300S<br>3684: 5’-GCGGCCATCTCGCAGATCATGAAGGAGCTG-3’<br>3685: 5’-CGCGACATCGCTTTGCACACGCTTCTGTAC-3’<br><br>Primers to mutate K296A/M297A<br>3686: 5’-GTGTGCAAAGCGATGTCGCGGCGGCCATCTCGCAGATC-3’<br>3687: 5’-GATCTGCGAGATGGCCGCCGCGACATCGCTTTGCACAC-3’<br>(Site-directed mutagenesis)
pcDNA4/ TO-KAP1 HP1 box mutant:F	V488E<br>[This study]	3398: 5’-GGCGTTCAAGGCTCTCTCGTGGCACCTTG-3'<br>3399: 5’-CAAGGTGCCACGAGAGAGCCTTGAACGCC-3'<br>(Site-directed mutagenesis)
pcDNA4/ TO-KAP1 NLS PM1:F	K469A/R470A<br>[This study]	3693:<br>5’-CCCCATGTGTCAGGTGTGGCAGCGTCCCGCTCAGGTGAGGGC-3’<br>3694:<br>5’-GCCCTCACCTGAGCGGGACGCTGCCACACCTGACACATGGGG-3'<br>(Site-directed mutagenesis)
pcDNA4/ TO-KAP1 NLS PM2:F	R483A/K484A<br>[This study]	3690:<br>5’-GAGGTGAGCGGCCTTATGGCCGCGGTGCCACGAGTGAGCCTT-3'<br>3691:<br>5’-AAGGCTCACTCGTGGCACCGCGGCCATAAGGCCGCTCACCTC-3'<br>(Site-directed mutagenesis)
pcDNA4/ TO-KAP1 NLS full mutant:F	K469A/R470A/R483 A/K484A<br>[This study]	Primers to mutate R483A/K484A<br>3690:<br>5’-GAGGTGAGCGGCCTTATGGCCGCGGTGCCACGAGTGAGCCTT-3'<br>3691:<br>5’-AAGGCTCACTCGTGGCACCGCGGCCATAAGGCCGCTCACCTC<br><br>Primers to mutate K469A/R470A<br>3693:<br>5’-CCCCATGTGTCAGGTGTGGCAGCGTCCCGCTCAGGTGAGGGC-3'<br>3694:<br>5’-GCCCTCACCTGAGCGGGACGCTGCCACACCTGACACATGGGG-3’<br>(Site-directed mutagenesis)
pcDNA4/ TO-KAP1 ΔIDR+NLS:F	KAP1 plus SV40 T-Ag NLS<br>[This study]	3626:<br>5’-GCCACCATTTGCCGTGTC-3’<br>3627:<br>5’-AGGGCCTGTTGAGTTAGTG-3’
pcDNA4/ TO-KAP1 NLS full	KAP1 with R483A/K484A plus SV40 T-Ag NLS	1107:<br>5’-CCGAAGCTTATGGCGGCCTCCGCGGCGGC-3’<br>3692:

mutant + NLS:F	[This study]	5'-CCGCTCGAGAACCTTACGCTTCTTTTTAGGTGGTGCACCTCCGCCGGGGCCATCACCAGGGCCAC-3'

Supplementary Table S2. Antibodies used in this study.

**Antibodies**	**Source**	**Identifier**	**Incubation time (temperature)**
Anti-KAP1 [20C1], Mouse monoclonal	Abcam	ab22553	1 hr (RT) to overnight (4°C)
Anti-Flag [M2], Mouse monoclonal	MilliporeSigma	F3165	1 hr (RT)
Anti-mouse IgG, HRP conjugated	Cell Signaling Technology	7076S	1 hr (RT)
hFAB Rhodamine Anti-Actin	Bio-Rad	12004163	1 hr (RT) to overnight (4°C)
Goat, anti-mouse AF 488	Thermo Fisher Scientific	A32723	1 hr (RT)
